# Genomics and Privacy: Implications of the New Reality of Closed Data for the Field

**DOI:** 10.1371/journal.pcbi.1002278

**Published:** 2011-12-01

**Authors:** Dov Greenbaum, Andrea Sboner, Xinmeng Jasmine Mu, Mark Gerstein

**Affiliations:** 1 Program in Computational Biology and Bioinformatics, Yale University, New Haven, Connecticut, United States of America; 2 Department of Molecular Biophysics and Biochemistry, Yale University, New Haven, Connecticut, United States of America; 3 Sanford T. Colb & Co. Intellectual Property Law, Marmorek, Rehovot, Israel; 4 Center for Health Law, Bioethics and Health Policy, Kiryat Ono College, Israel; 5 Center for Law and the Biosciences, Stanford Law School, Stanford University, California, United States of America; 6 Department of Computer Science, Yale University, New Haven, Connecticut, United States of America; University of California San Diego, United States of America

## Abstract

Open source and open data have been driving forces in bioinformatics in the past. However, privacy concerns may soon change the landscape, limiting future access to important data sets, including personal genomics data. Here we survey this situation in some detail, describing, in particular, how the large scale of the data from personal genomic sequencing makes it especially hard to share data, exacerbating the privacy problem. We also go over various aspects of genomic privacy: first, there is basic identifiability of subjects having their genome sequenced. However, even for individuals who have consented to be identified, there is the prospect of very detailed future characterization of their genotype, which, unanticipated at the time of their consent, may be more personal and invasive than the release of their medical records. We go over various computational strategies for dealing with the issue of genomic privacy. One can “slice” and reformat datasets to allow them to be partially shared while securing the most private variants. This is particularly applicable to functional genomics information, which can be largely processed without variant information. For handling the most private data there are a number of legal and technological approaches—for example, modifying the informed consent procedure to acknowledge that privacy cannot be guaranteed, and/or employing a secure cloud computing environment. Cloud computing in particular may allow access to the data in a more controlled fashion than the current practice of downloading and computing on large datasets. Furthermore, it may be particularly advantageous for small labs, given that the burden of many privacy issues falls disproportionately on them in comparison to large corporations and genome centers. Finally, we discuss how education of future genetics researchers will be important, with curriculums emphasizing privacy and data security. However, teaching personal genomics with identifiable subjects in the university setting will, in turn, create additional privacy issues and social conundrums.

**This is an “Editors' Outlook” article for *PLoS Computational Biology***

## The Current Situation in Bioinformatics: Tensions between Open Data and Limited Access

Bioinformatics' explosive growth over the past decades owes a lot to the open-source and open-data mentality of its practitioners. The biological sciences, and particularly computational biology and bioinformatics, have been driving forces in the development of data mining tools due, in part, to the availability of huge open data sets; this enormous amount of freely available data has become part of the ethos of genomics research. In contrast, in the social sciences, finance, and legal fields, large-scale data sets on the order of those found in bioinformatics are hard to find, and data is often sold rather than freely available.

Open-source software, such as software developed under the GNU license or operating systems such as Linux, was an original inspiration. It has allowed for the development of novel tools and code that can be improved, modified, and tweaked by subsequent users to precisely fit the current needs of individual researchers. Open source software was and continues to be used to build, maintain and mine databases that have greatly facilitated the development of bioinformatics research. Open data goes hand in hand with open source, as it is essential for the development and testing of open software tools. Much open data has been available to the bioinformatics community from a variety of databases, including the Protein Data Bank (PDB), a repository for macromolecular structures [1] (established 1971), and the National Center for Biotechnology Information (NCBI), which houses genomic sequences and other biotechnology-related information (1988) [2].

Open data not only provides the non-experimentalist with the necessary information to conduct analyses, but it allows for the replication and validation of previously published results. Further, sharing data allows for important nomenclature and terminology standards to be developed and refined, a necessity as data sets continue to get larger and more complex. The virtue in open data is so great that it has been become virtually a precondition for funding labs for genome sequencing. Moreover, several scientific journals require the data to be publicly available before accepting a manuscript for publication.

There has always existed, however, tension between the open-source/open-data movement, represented by academic bioinformatics, and those who would rather limit access. In some instances, those in favor of limited access are concerned about patient privacy—as is often the case in the medical fields. However, others have attempted to develop databases that were closed or of limited access to the basic science research public for commercial business purposes. This dichotomy was most dramatically presented in the initial sequencing of the human genome, which involved a “competition” between a public consortium, which favored the open-data approach, and Celera, a private company trying to develop a propriety database related to human genomics and claim associated intellectual property (IP) rights [3].

## The Future: More Closed Data?

Bioinformatics today is at a crossroads, and the pendulum is definitely swinging in favor of more limited access to data. This shift is happening for a number of reasons: First, the sheer size of the data makes readily transferring it and sharing it more difficult than it has been in the past. Secondly, the nature of the data is becoming more personally revealing, and is therefore considered more private and protectable. As we will describe, this more limited access to data is of particular concern to small labs and individual researchers.

With next-generation sequencers bringing down the cost of analysis faster than Moore's law (http://genome.gov/sequencingcosts/), data sets are becoming so large and unwieldy that it is often difficult to download and locally analyze relevant data; in the not-so-distant past, bioinformatics data were freely uploaded and downloaded using off-the-shelf, and/or lab-based web servers.

In addition to physical practical constraints on sharing, researchers may encounter an increased amount of IP protections and restrictions on data. These protections are experiencing a renewed emphasis as a result of efforts to commodify genomic information. IP protection is non-trivial and scientific data can be controlled, depending on the particular jurisdiction, under numerous different IP regimes, often simultaneously [4]. Although the legal issues surrounding this control are in flux and constantly evolving, and there remain broad discrepancies as to how bioinformatics will be legally protected around the world, the use of these protected datasets can still have significant legal repercussions, even for public research institutions.

## Privacy in Personal Genomics: Scale, Identification, and Characterization

In addition to the various IP protections, the handling of private and sensitive information that can now be collected with new sequencing technology necessitates additional levels of protection for data, further limiting its access. And, as genome sequencing reveals more about an individual, the distinction will be harder to make between medical records and human genome sequences.

In general, when collecting data from human subjects, it is important that each subject be fully informed of the experimental protocols and the data collected from those experiments, before giving their consent. Human subject protocols should be designed to minimize the potential of harm to the subject, while maximizing the potential benefits. For example, post-data collection: (i) protections must be in place to prevent unauthorized access to human subject data; (ii) access must be restricted to those individuals with a legitimate research interest in the data, who also must understand how to properly handle the data to keep it out of the wrong hands; and (iii) data must also be properly disposed of when no longer needed. For digital data, the information technology (IT) administrators of the systems on which this data is stored have a responsibility to maintain strong IT security policies, and keep the systems fully patched, with up-to-date antivirus definitions, for example.

The above text is fairly generic, and many of the issues have already been broached with the development of electronic patient records that collect, store, and share medical data typically across health care operators. A comprehensive set of rules and regulations have already been promulgated to ensure that sensitive information is accessed only by authorized people, and with the final goal to improve the quality of care [5].

However, the nature of the current data sets being churned out requires a different approach.

First, there is the scale of the data. It is much more difficult to deal with terabytes of encrypted data in the framework of large calculations than it is to deal with a small amount of encrypted text in a medical record meant to be read by humans. To do proper in-depth processing of next-generation sequencing data, conventional encryption becomes rather cumbersome and difficult.

Second, the identification of DNA sequence variants can readily act as a source of identifiable information. In particular, a minimum number of 75 independent SNPs, if not fewer, will uniquely identify a person, albeit without being able to phenotype that individual with the limited SNP data [6]. However, the degree to which DNA data is identifiable is not always obvious. Therefore, until recently, given the onerous requirements for explicit consent for each individual's data set [7], approaches were developed to facilitate research on these data sets via de-identification of patient information [8]. The data treated this way has been made publicly available in the past and has further facilitated discoveries in medical research.

It has recently been shown, however, that it is even possible to re-identify genotyped individuals or even individuals in pooled mixtures of DNA [9]. Once re-identified, this gives rise to the potential for the revelation of significantly personal information, regarding the formerly anonymous source. This prompted the United States National Institute of Health (NIH), the Broad Institute in the US, and the Wellcome Trust in the United Kingdom to further restrict the access to the data from genome-wide association studies.

The risk of identification comes from multiple possible different sources. In general, while data sets in genomics research can be anonymized they often need not be, depending on the wishes of the patients. Thus, on a simple level, some patients will opt to provide their DNA without any preconditions. In other instances, patients will consent to have their DNA analyzed but will insist on not being identified. And, in between these extremes, subjects will provide DNA without restriction provided that it be used only for a particular direction of research, but may limit the usage of the DNA for say, research into a disease with an attached stigma.

Further, there are many instances where one might gain access to DNA to cross reference with a publicly available data set. These include, but are not limited to: (i) surreptitiously obtaining DNA from a discarded personal item; (ii) other public or private DNA databases such as those kept by law enforcement; (iii) biological samples from medical procedures; (iv) DNA samples from close relatives; or, (v) one's own DNA in determining a biological parent.

Third, it is important to distinguish between the issues of identification and of characterization. Even if subjects consent to revealing their identity, they may not have consented to detailed characterization. That is, a consenting subject might not realize how much information is being given away by genome sequencing. Those who subscribe to the notion of genomic exceptionalism, i.e., that genetic data is somehow categorically different than other forms of medical information, in particular, note that genomic data is much more informative than standard medical records as it can provide risk-related data pertaining to medical and non-medical conditions across family trees, including risks of future illness, undiagnosed psychiatric conditions, and even physical traits. And while we cannot currently fully interpret it, we will soon be able to, and once this information is published it cannot be taken back. Furthermore, the fact that children carry half the genetic information of their parents implies that a decision to reveal one's genetic information today has repercussions for generations to come.

## How Difficult Is It to Deal with Private Data Sets?

Under the current open-data regime, bioinformatics investigators can directly access the data hosted by repositories such as Gene Expression Omnibus (GEO) [10], ArrayExpress [11], GenBank [12], or use free web tools such as Ensembl [13] or the UCSC GenomeBrowser [14]. Effort from the final user viewpoint is limited to transfer time and the size of the data set. The burden on big research centers as well as small labs is equivalent and nearly non-existent.

Current human genomics data—in particular, readouts from next-generation sequencing—contains a lot of information that can, in principle, both identify and characterize an individual. Hence, in this context, these data need to be properly managed, and accessing them requires proper controls. To illustrate the impact of closed data in a traditionally open world, we describe aspects of the interaction with the database of Genotypes and Phenotypes (dbGaP) [15] and the International Cancer Genome Consortium (ICGC) [16]. Both provide excellent examples on how to properly handle private information for research studies. However, access to the private part of the databases is far from “click-and-download”, which many researchers are used to. Access often requires institutional review board (IRB) authorization. For example, ICGC requires IRB authorization prior to the submission of the application to access and download the data. Typically, this entails a description of the requested data, the management of the data on the user's site (e.g., for digital data, security levels of stored data, and the list of authorized people accessing them), and the type of analysis. This process can take several weeks or a few months depending on the institution. Moreover, if the researcher wants to perform additional analyses that were not foreseen at the time of the first IRB authorization, due for example to advances in computational algorithms, or to the availability of new data sets allowing for integrated analysis, a second authorization may be required. Although these restricted controls satisfy the need for privacy protection of the data, the administrative burden may limit their accessibility to only those who really take the effort to access them. When possible, researchers may prefer to use freely available genomics data. An example of such freely available data is that provided by the Personal Genome Project (PGP). For example, since fall 2008, more than 34,000 investigators have viewed the genomic data of the first PGP individual, i.e., a rate of ∼1,000 per month per data set (PGP-1, personal communication). This is in contrast to the ICGC, for example, where only seven projects (as of October 2011) have been approved for access to the controlled data since December 2010 (http://www.icgc.org/daco/approved-projects/), i.e., ∼0.00023 per month per data set, and another seven are being currently revised (J. Jennings, personal communication). Although it is expected that more projects will be approved by ICGC in the future, this difference with open data is striking.

Furthermore, not only does accessing and downloading the data entail a considerable effort for end users, but also making genomic data available to the research community can be quite cumbersome, requiring substantial paperwork. The whole process is disproportionally onerous for small labs, which may not have the proper experience or resources for the submission of one or two data sets.

The administrative efforts to access private genetic data exact a real cost and create a drag on research efforts creating friction in the depositing, accessing, and analyzing of data. With many academics risk averse and cost conscious the time and effort often necessary to access this data will cut down on potential research efforts.

## Computational Approaches to Dealing with Private Data

Given how difficult it is to handle large amounts of private data, one can imagine a number of computational approaches to ease the burden. First, one can try to “slice” out some of the relevant variants in a big data set, i.e., selectively releasing SNPs and other genomic variants, such as small indels and larger structural variations, that are proximal to a known locus of interest (e.g., related to a disease). Alternatively, more extensive filtering of genomic variants may involve other genomic properties (such as heterozygosity and allele frequency) and its immediate sequence context (such as proximity of a recombination hotspot and the local sequence conservation level). Furthermore, one may consider to only release the summary statistics from genomic property calculations over sliding windows across the genome, such as the average allele frequency and number of variants. A final idea involves building “synthetic” personal genomes from a pool of individual genomes in a group. To be more specific, one may permute the variants or variant blocks between individual genomes, such that the representative variations of the entire group are readily seen, but not those of any particular individual. While the exact manipulation of the variation annotation file could be done in a reversible and uniquely determined way, using a key private to the researcher, persons without the key would not have adequate information to recover the data.

However, one should keep in mind that although this reduces the public exposure of the sequences, none of these data manipulation methods fully de-identifies the test subjects. These methods should rather be viewed as options to mask part of a personal genome, preventing some aspect of detailed characterization. Nonetheless, even at this point, it is not sensible to completely rule out the possibility of the sequencing data being deciphered. Using sufficiently sophisticated statistical models given the prevalence of linkage disequilibrium (LD) in the human genome, the sequencing data from a personal genome may be decoded eventually from haplotypes in the human population to a high accuracy by persons with specialized knowledge of population genetics. A particularly famous example of this is the determination of Jim Watson's apoE genotype. He explicitly did not want this revealed in his personal genome sequencing because of its implications related to mental disease. However, researchers showed that the initial amount of sequence masked was not sufficient to hide the key variant if one took into account LD [17].

Second, one can try to anonymize functional genomics data; increasingly, the readout of many functional genomics experiments is in the form of sequencing. Privacy concerns relate to functional genomics data differently from genome-sequencing data. Specifically, when the low-level sequencing data is released, one can essentially recover the genomic variations in a similar fashion as in genome-wide sequencing. Nonetheless, these genomic variations are only limited to the corresponding functionally annotated regions (∼5% of the human genome). Hence, naively, one may get the impression that genomic privacy protection is less critical for functional genomics data. However, all experiments on humans essentially give rise to variant information; effectively, an RNA-Seq experiment is almost equivalent to exome sequencing.

However, unlike genome sequence, the variants are not always the key information revealed by an experiment.

In particular, if only the high-level data—such as the ChIP-seq peak intervals and RNA-seq gene expression levels, are submitted, the DNA-level genomic variations (i.e., SNPs) are, to a large extent, masked. Therefore, the concerns for genomic privacy are minimal. For example, RNA-seq expression values are almost equivalent to expression microarray data, which have not posed any privacy issues. Indeed, microarray data have been publicly available for some time via repositories such as GEO [10] or ArrayExpress [11]. Given this, in some cases, it is possible to reduce the impact of sequencing information via simple data manipulation. For example, RNA-Seq experiments measure the transcriptome of a population of cells [18]. Typically, the main goal of these investigations is the identification of differentially expressed genes, isoforms, or exons between different conditions. This type of analysis can be carried out without including explicit sequence variants, thus greatly reducing the potential identifiable information, although there will still be some identification issues. Theoretically, the pattern of expression levels measured by a sequencing experiment may still lead to the identification of the individual. However, this possibility is also shared by gene expression and exon microarrays that have been freely shared in the past via public repositories. RSEQtools proposes Mapped Read Format (MRF) as a practical realization of this. MRF is a compact data format that can separate the alignment and genomic “signal” information from the actual sequences [19]. This separation has the advantage to effectively allow a fine-tuned access control to the data, by making the alignment data publicly available, whereas the sequences may be kept under restricted access. It also has the advantage of providing compact data sets, especially now with increasing sequence read lengths that can be publicly shared.

Another approach may become a popular archival format for reducing the size of next-generation sequencing data is reference-based compression and the associated CRAM format [20]. This format stores the position of a read on a reference and then the variations in the read relative to the reference. If the read cannot be mapped to the reference, one makes up a rough assembly on the fly and then maps the read to this. This format can be readily adapted to anonymize information in a similar fashion to MRF. One simply just stores the first bit of information, the position that the read maps onto the reference, and leaves off the remainder of the information (the variants on the read relative to the reference which constitutes sensitive information).

The approach taken by MRF for RNA-seq can be easily adopted by other functional genomics experiments, such as ChIP-seq. Here, the locations of the peaks typically constitute sufficient data summaries for the downstream analysis. Again, separating the sequences from the alignment has the advantage to create a two-tier environment, one public and one private, that can satisfy both the privacy requirements as well as the sharing of the data to the research community.

## Approaches to Future Data Management: No Confidentiality, Banking Models, and Private Clouds

Many of the complexities of dealing with private and large-scale information disproportionally burden small laboratories. They do not have the staff to secure the computers, encrypt the data, and deal with all the forms and approvals necessary that large genome centers in big companies have. Then how can they profitably engage in medical research using large-scale private data?

One extreme approach would be to have no privacy at all in genomic data for medical research. That is, we would not make any pretense in trying to protect genomic information and only seek volunteers who would consent to have their information be publicly available. This is an ethically honest but extreme approach to consent [21].

It has been adopted by the PGP [22]. The PGP has been so far very successful with the number of the early individuals in the project garnering a considerable amount of publicity and having their sequences viewed quite a bit. However, it's not clear that this approach would scale to potentially millions of people who will be having their genome sequenced. It is essentially asking the sequenced individual to be a test pilot for scientific research, risking their privacy to advance the frontier.

Another approach could be to learn from the legal and banking sectors wherein privacy and confidentiality are protected while the practitioners nevertheless manipulate and analyze large databases of highly confidential personal and financial data. Furthermore, private information is exchanged between many organizations ranging from large companies to small law firms. In those cases, incentives to keep clients, as well as governmental regulations with stiff penalties and civil and criminal repercussions, help to prevent breaches of customer privacy.

An aspect of the legal and financial model is accreditation and licensure, which requires practitioners to show proficiency in their craft and in the legal and social concerns. Licensing also creates liability, creating real world repercussions, i.e., penalties and/or forfeiture of the license and their ability to access the data, in the event of a breach of responsibilities. Licensure could follow the example of the legal profession, where local and national organizations bear the responsibility of licensing, and wherein national organizations can accept the credentials of those licensed elsewhere.

However, there are some key differences between the legal and financial approach and that required by academia. There is no incentive to publish and share information in the private world of banking as there is in academia. Furthermore, most of the individuals involved with private information are not student trainees, but rather informed professionals.

A third potential solution might be a government-supported cloud computer repository. There are a number of clear advantages to cloud computing [23]. It can provide a centralized and relatively homogeneous interface for genomic researchers. It can provide the computational power and memory to allow for the manipulation of these large data sets off-site—something that smaller labs or individual researchers may not otherwise have access to. Further, by having the data centralized by a government or large entity, economies of scale allow the necessary security and precautions to protect private and/or proprietary data to be universally employed. Whereas many labs without the financial or technological wherewithal may have previously just posted their results on a local server, the cloud potentially may be able to provide universal protection to data at a standard heretofore affordable only to large labs.

Instead of risking privacy every time a researcher downloads a data set, analytical programs to access and analyze the data can be uploaded to the cloud where the programs can not only analyze the data, but can also be shared and improved upon by others. Most importantly, what happens in the cloud, while nevertheless staying in the cloud, cannot be hidden—all access to the data and the nature of the access can be logged and reviewed to prevent abuse of the data and breaches of privacy. Researchers will be unable to just take data off the servers, and the massive size of most files mean that data cannot simply be copied by hand; rather, the cloud infrastructure will necessitate that a recordable event occurs wherein a researcher downloads or possibly even views a file onto their own system.

This logging system is necessary not only because of the extremely unlikely event of malicious actions by researchers, but more importantly, to prevent students, who may be unaware of the greater repercussions of their actions, from accidentally and innocuously breaching privacy and security, i.e., a naive but errant Perl script that inadvertently sends private data onto the Internet.

## Summary and Direction: Educating Researchers about the New Reality

Clearly, with the advent of large amounts of personal genomic data, bioinformatics is in for a change. It is inevitable that much of this data will not be open in the way we have grown accustomed to in the past. This is going to necessitate new approaches to anonymizing data sets and providing secure computational environments. It will also require us to educate a new generation of researchers to think more carefully about personal genomics and privacy.

In an effort to inculcate young researchers regarding the ramifications of genomic sciences, numerous universities have recently implemented programs to provide some students—in the extreme, the entire incoming freshman class—access to personal genomic technologies. These efforts, however, raise pedagogical and social concerns.

The underlying goal of each of these programs seems to be to introduce and to acclimate young adults to what is likely to be a common, prevalent, and relevant technology in the future. However, these programs are also a double-edged sword. The Facebook/Twitter generation, in particular, has an evolving concept of personal privacy that may not be compatible with how society currently perceives the nature of the information provided to them by personal genomics.

Further, one of the most effective ways in these programs to educate young people about genomics is to have them study their own, their relatives', and even their peers' genomes. This is, of course, what the students can most easily relate to. However, it is also the type of information that has one of the greatest degrees of privacy implications: there are concerns for the student's own privacy—in the extreme, students, desensitized to privacy concerns, may post their genetic results publicly—and there are additional concerns for the student's extended families that share much of their genetic information, and may or may not consent to having their genetic predispositions aired publicly.

There are further real concerns that students, provided with powerful genetic information (e.g., Alzheimer's predispositions) may fail to adequately protect it. Curious students are likely to seek and search out this most intriguing data, instead of the more pedestrian data regarding eye color or propensity to develop wet earwax. Unfortunately, the most interesting data will always be the data that requires the most protections.

Further, not withstanding privacy concerns, restrictions on access to this powerfully pedagogical data may limit the usefulness of the educational exercise or invite curious students to circumvent what may be in many cases purposeful limitations on access.

Open access to research data, once a given in genomic research, is becoming rarer, and privacy concerns regarding current and future genomics research data present a further non-trivial obstacle to data sharing; finding an optimal balance between access for researchers and protection for patients' privacy remains elusive. Here, we have provided a survey of the current situation, noting in particular how the large-scale data from next-generation sequencing makes it especially hard to share data, exacerbating privacy and open-data problems. We presented various computational strategies for dealing with the issue of genomic privacy, and note how cloud computing potentially may allow access to the data in a more controlled fashion than the current practice of downloading and computing on large data sets, perhaps helping to reverse the trend against open data.

**Authors' Biographies**

**Dov Greenbaum** is licensed to practice in California and before the United States Patent and Trademark Office. Dov has a JD from the University of California, Berkeley, and a PhD in Genetics from Yale University.

**Andrea Sboner** was an Associate Research Scientist in Computational Biology and Bioinformatics at Yale University with a main focus on the processing and analysis of next-generation sequencing experiments. Currently, he is an instructor at the Department of Pathology and Laboratory Medicine and at the Institute for Computational Biomedicine, Weill Cornell Medical College.

**Xinmeng Jasmine Mu** is a doctoral student in Computational Biology and Bioinformatics at Yale University.

**Mark Gerstein** is the A. L. Williams Professor of Biomedical Informatics at Yale University, where he co-directs the Yale Computational Biology and Bioinformatics Program. His laboratory uses computation to annotate genome sequences, mine data on gene expression and molecular networks, analyze protein families, and simulate macromolecular structures. A former W. M. Keck Foundation Distinguished Young Scholar, he received his PhD at Cambridge University.
